# Cryptogenic Perirolandic Brain Abscess in an Otherwise Healthy Young Man

**DOI:** 10.1002/ccr3.72056

**Published:** 2026-04-23

**Authors:** Mazen Taman, Carl Porto, Jean‐Luc Lewis, Dimitrios Farmakiotis, Athar N. Malik

**Affiliations:** ^1^ Department of Neurosurgery Warren Alpert Medical School of Brown University Providence Rhode Island USA; ^2^ Division of Infectious Diseases, Beth Israel Deaconess Medical Center Harvard Medical School Boston Massachusetts USA

**Keywords:** *Aggregatibacter aphrophilus*, brain abscess, cryptogenic brain abscess, ring‐enhancing lesion, stereotactic aspiration, *Streptococcus intermedius*

## Abstract

A previously healthy 19‐year‐old male presented with 1 day of transient right‐sided weakness, numbness, and gait disequilibrium after recent self‐limited sinonasal symptoms and minor nasal trauma with epistaxis. He was afebrile but with focal deficits, leukocytosis, and elevated C‐reactive protein. Noncontrast head CT showed a left frontal lesion with vasogenic edema; MRI demonstrated a 2.4 × 1.4 cm rim‐enhancing lesion in the left paramedian precentral gyrus with homogeneous diffusion restriction, low apparent diffusion coefficient, and surrounding edema. Stereotactic aspiration was performed within 24 h of presentation to obtain microbiological diagnosis and source control. A neuronavigated, transgyral frontal approach was used to avoid eloquent cortex. Empiric antimicrobials were initiated immediately after operative sampling. Postoperatively, the patient developed transient distal right lower extremity weakness that improved with supportive care and physical therapy. A thorough cardiac, dental, and thoracic evaluation did not reveal a source; the case was deemed cryptogenic. Operative cultures grew *Streptococcus intermedius* and *Aggregatibacter aphrophilus* . Targeted intravenous therapy was administered with near‐complete radiographic resolution and full neurologic recovery at 4‐month follow‐up. This case emphasizes three practical principles for trainees: maintain suspicion for intracranial infection in afebrile young patients with new focal deficits and recent sinonasal symptoms; prioritize microbiological diagnosis and source control, while not delaying empiric therapy; and plan stereotactic trajectories that respect eloquent cortex and anticipate transient postoperative deficits. Early imaging, meticulous source evaluation and control, and directed therapy remain central to outcome.

## Key Clinical Message

In young, afebrile patients with new focal deficits, maintain suspicion for brain abscess even without an obvious source. Suspected brain abscess warrants rapid imaging, timely neurosurgical drainage for microbiological diagnosis and source control (ideally within 24 h), and prompt empiric antibiotics. Reserve “cryptogenic” until dental, cardiac, and thoracic evaluations are negative.

## 1 Introduction

Brain abscess is an uncommon but high consequence diagnosis, with an estimated annual incidence of approximately 0.2–1.9 per 100,000 in contemporary series, and reported mortality of 17%–32%, particularly high with intraventricular rupture [1, 2]. In the United States, abscesses account for approximately 1%–2% of intracranial masses, more frequently affecting males, and most commonly localizing to the frontal and temporal lobes [1]. These infections arise via hematogenous seeding, contiguous spread from sinuses, middle ear, or dentition, or less commonly direct inoculation after surgery or trauma [3]. The most commonly isolated organisms are *Streptococcus* species, particularly *Streptococcus intermedius* , followed by *Staphylococcus aureus* and anaerobes; *Nocardia* and fungi are more typical in immunocompromised individuals [4]. Even with thorough evaluation, up to 20%–30% of brain abscesses have no demonstrable source and are considered cryptogenic [4].

## 2 Case History

A 19‐year‐old previously healthy male presented to the emergency department with 1 day of transient right‐sided numbness, weakness, and gait disequilibrium. He had no fever or headache. Two weeks prior to presentation, he and his mother experienced self‐limited upper respiratory symptoms consistent with sinusitis. One week prior to presentation he sustained nasal trauma during basketball practice with significant epistaxis followed by brief clear rhinorrhea. The day before presentation he was struck on the occiput by a basketball, and his neurologic symptoms developed a few hours later.

## 3 Differential Diagnosis, Investigations, and Treatment

**FIGURE 1:**
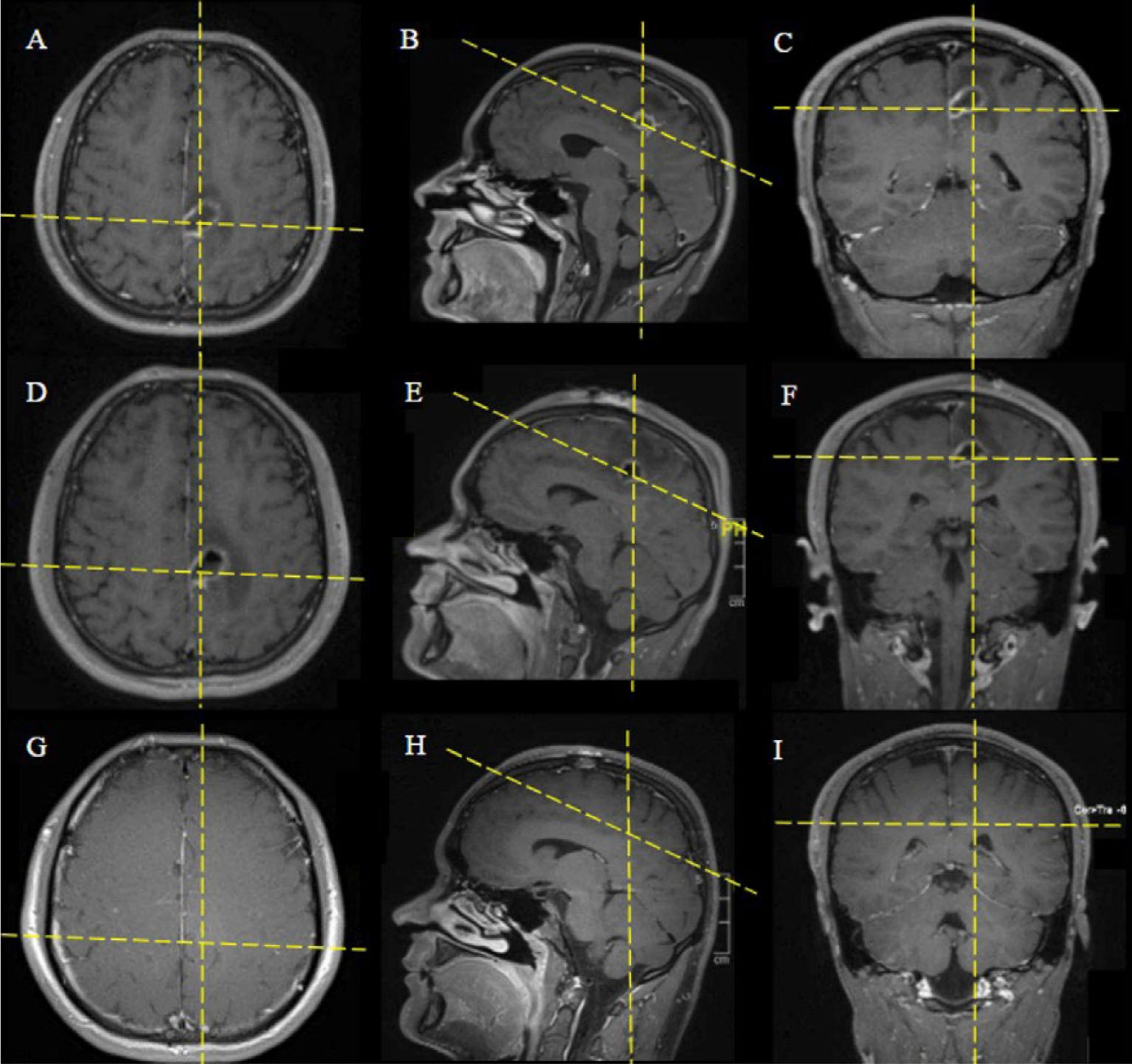
MRI evolution of left perirolandic paramedian brain abscess (T1‐weighted fat‐saturated post‐contrast): Preoperative axial/sagittal/coronal (A–C) show a 2.4 × 1.4 cm thin rim–enhancing lesion with surrounding vasogenic edema. Postoperative day 1 (D–F) demonstrates persistent rim enhancement with slightly increased edema. Four‐month follow‐up (G–I) shows near‐complete resolution with only minimal residual enhancement.

On arrival he was afebrile and hemodynamically stable with transient right‐sided weakness, numbness, and gait disequilibrium. Laboratory testing revealed leukocytosis (10.6 × 10^9^/L, reference range: 4.2–10.0 × 10^9^/L) and elevated C‐reactive protein (11.94 mg/L, reference range: 0.00–10.00 mg/L). Noncontrast CT scan of the head was obtained within 2 h of presentation and demonstrated a left frontal lesion with surrounding vasogenic edema, without evidence of facial or skull base fractures. MRI of the brain, completed within 5 h of presentation, showed a 2.4 × 1.4 cm ring‐enhancing lesion in the posterior left frontal paramedian cortex, characterized by central diffusion restriction, prominent surrounding edema, and sulcal effacement, features most consistent with a brain abscess (Figure 1).

**FIGURE 2:**
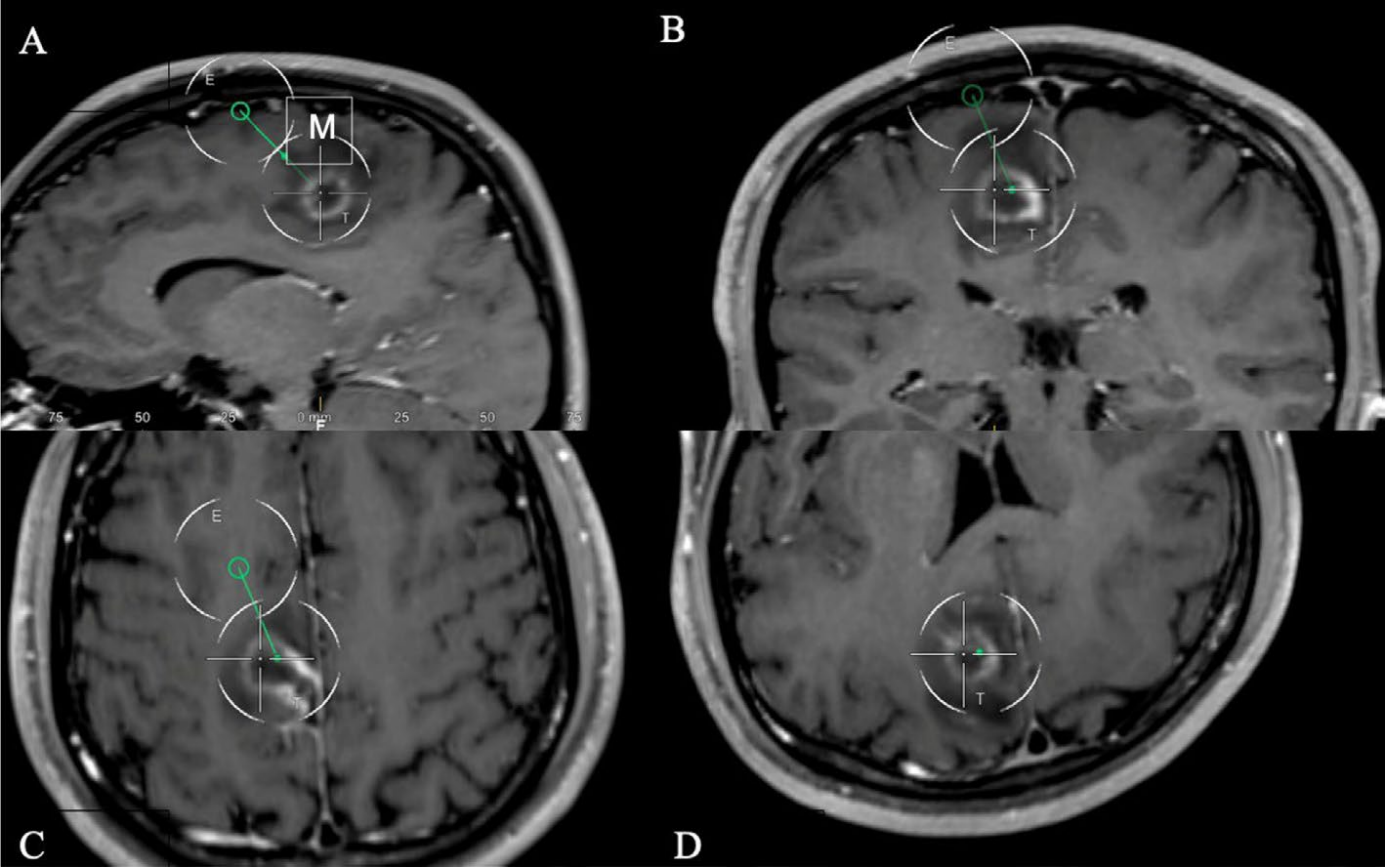
Preoperative planning of the surgical trajectory for aspiration of the abscess. The green circle represents the entry point of the aspiration needle, and the intersecting lines represent the target for aspiration. The letter “M” denotes the motor cortex. The trajectory is demonstrated in the (A) sagittal, (B) coronal, (C) axial, and (D) perpendicular axis on a T1‐weighted fat‐saturated, post‐gadolinium, contrast‐enhanced MRI.

Given the absence of systemic signs of infection and anticipated urgent drainage, empiric antibiotics were withheld for a brief interval to preserve the diagnostic yield of stereotactic aspiration. The abscess was located near the posterior middle frontal gyrus, just anterior to the motor cortex. He underwent a neuronavigated, transgyral left frontal stereotactic aspiration to minimize disruption of eloquent tissue (Figure 2). Aspiration was performed within 24 h of presentation and 20 h of MRI diagnosis, and frank pus was obtained and sent for culture. Empiric antimicrobial therapy was initiated immediately after sampling and subsequently narrowed based on culture results.

Postoperatively, he developed new right foot weakness, attributed to local edema near the motor strip. Strength improved steadily with supportive care and physical therapy. Inpatient evaluation found no odontogenic disease, chest imaging was unremarkable, and transesophageal echocardiography showed no endocarditis or intracardiac shunt. Given persistent diagnostic uncertainty, we considered occult right‐to‐left shunt; high‐resolution, contrast‐enhanced chest CT found no pulmonary arteriovenous malformation. In the absence of a convincing sinonasal, odontogenic, cardiac, or thoracic source, the abscess was deemed cryptogenic.

## 4 Conclusions and Results

Cultures grew *Streptococcus intermedius* and *Aggregatibacter aphrophilus* . He completed 6 weeks of intravenous ceftriaxone. By 4‐month follow‐up, MRI showed near‐complete resolution of the abscess with full return of strength.

## 5 Discussion

This case illustrates a rare polymicrobial brain abscess in a previously healthy young adult, with cultures growing *Streptococcus intermedius* and *Aggregatibacter aphrophilus* —an unusual pairing with only three cases previously reported in the literature and no prior cases reported in the United States. While the patient reported minor nasal trauma and was later struck on the head, there was no radiographic evidence of fracture or direct inoculation. Given that *S. intermedius* and *A. aphrophilus* are common oral cavity organisms, hematogenous seeding from an unrecognized mucosal source remains plausible; however, in many patients, the specific route to the brain cannot be established. In this case, recent sinonasal symptoms and minor trauma may have been temporally associated but do not prove causation, particularly in the absence of radiographic evidence of skull base injury or direct inoculation. Sinusitis is implicated in roughly 25% of brain abscesses, whereas trauma—typically penetrating or with skull base involvement—accounts for a substantially smaller fraction (~9.3%) [5].

Extensive evaluation, including transesophageal echocardiography and dental evaluation, excluded other common routes of entry such as cardiac shunting and odontogenic infection [6, 7]. Another underrecognized etiology is pulmonary arteriovenous malformations (PAVMs), which permit septic emboli to bypass the pulmonary capillary filter and reach the cerebral vasculature [8]. These are most commonly associated with hereditary hemorrhagic telangiectasia but can occur sporadically [8]. In young patients with brain abscess and no identifiable source, contrast‐enhanced CT or CTA of the chest may reveal a silent PAVM. A pragmatic approach is to reserve the label “cryptogenic” until dental/ENT assessment, blood cultures, echocardiography (ideally with shunt evaluation), and contrast‐enhanced chest imaging fail to localize a nidus. When the history suggests recent sinonasal symptoms without objective source, cryptogenic classification remains appropriate and guides follow‐up rather than altering acute management.

Importantly, an apparently reassuring bedside examination may miss early neurocognitive sequelae, reinforcing that suspected brain abscess should be managed urgently even when patients appear well. Although headache, fever, and altered mental status are classic features, systemic signs of infection are absent in many patients [7]. Focal neurologic deficits may predominate, particularly in lesions affecting eloquent cortex. Involvement of the motor strip, as in this patient, commonly produces limb weakness; in one series, up to 65% of patients had deficits at discharge, with approximately two‐thirds improving by 1 year [9].

Neuroimaging remains the cornerstone of diagnosis. CT is widely accessible and useful for identifying mass effect and ring‐enhancing lesions, but MRI offers superior sensitivity and specificity [6] (Table 1). Mature abscesses typically exhibit ring enhancement with surrounding vasogenic edema and central diffusion restriction on DWI sequences [6]. Proton magnetic resonance spectroscopy (H‐MRS) can further distinguish abscess from necrotic tumor by identifying amino acids and metabolic byproducts such as lactate, acetate, and succinate [1]. These features were present in our patient and guided a neuronavigated trajectory just anterior to the motor cortex.

**TABLE 1:** Imaging characteristics of various intracranial masses, comparing brain abscesses, necrotic tumors, cystic metastases, and hemorrhagic infarcts [3, 10, 11, 12, 13]. Differences in ring enhancement, DWI, ADC, surrounding edema, mass effect, contrast response, and lesion borders aid in distinguishing brain abscesses from other lesions, supporting accurate diagnosis and treatment planning.

Characteristic	Brain abscess	Necrotic tumor	Cystic metastasis	Hemorrhagic infarct
Ring enhancement on CT/MRI	Smooth, thin, and uniform ring enhancement	Irregular, thick, and nodular ring enhancement	Irregular, thick, and nodular ring enhancement	Thin, incomplete ring enhancement
Diffusion‐weighted imaging (DWI)	Restricted diffusion (hyperintense on DWI)	No or mildly restricted diffusion	No restricted diffusion	No restricted diffusion in chronic phase
Apparent diffusion coefficient (ADC)	Hypointense on ADC	Variable ADC signal, often not as hypointense as abscess	Increased ADC signal	Variable ADC signal, often increased
Surrounding edema	Significant vasogenic edema surrounding the lesion	Mild to moderate edema	Mild to moderate edema	Mild to moderate edema
Mass effect	Often present, varies with abscess size	Variable, may be present	Present, often significant	Present in the acute phase
Response to contrast	Typically, strong and uniform enhancement	Variable, often heterogeneous enhancement	Variable enhancement	Variable, often mild enhancement
Lesion border characteristics	Irregular but smooth, well‐defined border	Irregular, poorly defined border	Irregular, often poorly defined border	Irregular and incomplete border

*S. intermedius* is a well‐established cause of brain abscess and frequently associated with sinus, dental, and pulmonary infections [4]. *A. aphrophilus* , a HACEK organism, is a less common cause of brain abscess, often reported in association with endocarditis, cyanotic congenital heart disease, or dental manipulation [14, 15]. Coinfections with *A. aphrophilus* and *S. intermedius* in polymicrobial brain abscesses are rare with only three cases reported in the literature, and none documented in the United States [16]. One case reported a 6‐year‐old female who had undergone dental filling 5 months prior to diagnosis, whose abscess was managed with stereotactic biopsy and 12 weeks of cefotaxime and metronidazole. A second case reported a 4‐year‐old male born with several congenital heart defects, including a ventral septal defect, whose abscess was managed with two aspiration procedures and 6 weeks of intravenous meropenem therapy [17]. The last report described a 56‐year‐old male with a remote history of atrial septal defect closure and dental procedures 5 months prior to diagnosis whose abscess was managed with aspiration and 8 weeks of ceftriaxone, metronidazole, and rifampin [18]. Our case contributes to this limited literature by documenting this pairing in an immunocompetent host without congenital cardiac disease or recent invasive dental procedures.

Prompt initiation of antimicrobial therapy is imperative to achieve favorable outcomes, as evidenced by a reported 50% increase of mortality risk per day of delayed antimicrobial administration [19]. Accordingly, empiric therapy should not be delayed when patients are toxic, neurologically deteriorating, or when drainage cannot be performed urgently. However, starting antibiotics before stereotactic aspiration of the abscess may compromise the diagnostic yield of the aspirated specimen. Therefore, in carefully selected patients in whom drainage is expected within 24 h, a brief deferral of antibiotics in order to obtain microbiological diagnosis may be reasonable [6, 20]. Empiric coverage commonly includes a third‐generation cephalosporin combined with metronidazole, or meropenem as an alternative; vancomycin should be added if *Staphylococcus aureus* is suspected [6]. Treatment options for *S. intermedius* include many beta‐lactams, clindamycin, and some fluoroquinolones, while *A. aphrophilus* typically responds to third‐generation cephalosporins and beta‐lactam/beta‐lactamase inhibitor combinations [4, 6, 14].

The typical duration of intravenous antimicrobial therapy for brain abscesses is 6–8 weeks, and can vary depending on neurological status and imaging findings, such as abscess size [6]. Cranial imaging should be repeated immediately if clinical deterioration occurs, at 1–2 weeks if there is no improvement, and then biweekly for up to 3 months until clinical recovery is confirmed [6].

Surgically, lesions greater than 2.5 cm in diameter, those with mass effect, or diagnostic uncertainty warrant drainage [1, 6]. Stereotactic aspiration is preferred for deep or eloquent locations and can be repeated if needed [1]. Craniotomy and excision are reserved for larger, multiloculated abscesses near the cortical surface, fungal abscesses, or those refractory to aspiration or antimicrobial therapy [6]. In our patient, stereotactic aspiration near the posterior middle frontal gyrus achieved source control and diagnostic yield, and the patient recovered fully with culture‐directed ceftriaxone.

This case of a polymicrobial cryptogenic brain abscess in an immunocompetent young adult underscores three practical lessons for trainees and clinicians: keep brain abscess on the differential in afebrile patients with new focal deficits and recent sinonasal symptoms; prioritize microbiological diagnosis and source control, while not delaying empiric therapy and plan stereotactic trajectories that respect eloquent cortex, anticipate transient deficits, and counsel patients accordingly. Although the organisms are not exotic, their co‐isolation highlights the polymicrobial nature of many abscesses and the rationale for broad empiric coverage with timely de‐escalation. Early imaging, meticulous source evaluation, and culture‐guided therapy remain the cornerstones of care. Vigilance in similar low‐risk presentations can accelerate diagnosis, personalize treatment, and improve outcomes.

## Author Contributions

**Mazen Taman:** conceptualization, investigation, methodology, resources, resources, visualization, visualization, writing – original draft, writing – original draft, writing – review and editing, writing – review and editing. **Carl Porto:** methodology, project administration, visualization, writing – original draft, writing – review and editing. **Jean‐Luc Lewis:** methodology, visualization, writing – original draft. **Dimitrios Farmakiotis:** project administration, resources, supervision, visualization, writing – review and editing. **Athar N. Malik:** conceptualization, methodology, project administration, resources, supervision, validation, visualization, writing – review and editing.

## Funding

This work received no specific grant from any funding agency in the public, commercial, or not‐for‐profit sectors.

## Consent

Written informed consent for publication of the clinical details and images was obtained from the patient. A copy of the consent is available for review by the journal editor upon request.

## Conflicts of Interest

The authors declare no conflicts of interest.

## Data Availability

The authors have nothing to report.
